# Trends in Adherence to the *Physical Activity Guidelines for Americans* for Aerobic Activity and Time Spent on Sedentary Behavior Among US Adults, 2007 to 2016

**DOI:** 10.1001/jamanetworkopen.2019.7597

**Published:** 2019-07-26

**Authors:** Yang Du, Buyun Liu, Yangbo Sun, Linda G. Snetselaar, Robert B. Wallace, Wei Bao

**Affiliations:** 1 Department of Epidemiology, College of Public Health, The University of Iowa, Iowa City

## Abstract

**Importance:**

The updated 2018 *Physical Activity Guidelines for Americans* (PAG) reaffirmed key recommendations regarding aerobic activity in the 2008 edition and recently introduced health risks of sedentary behaviors and their association with physical activity.

**Objective:**

To examine the concurrent changing trends in adherence to the PAG for aerobic activity and time spent on sedentary behavior in US adults from 2007 to 2016.

**Design, Setting, and Participants:**

This study used data from a series of cross-sectional, nationally representative surveys on adults 18 years or older from the National Health and Nutrition Examination Surveys from 2007 to 2016. Data analysis was performed from September 1, 2018, to March 31, 2019.

**Main Outcomes and Measures:**

Duration, frequency, and intensity of leisure-time, work-related, and transportation-related aerobic activity were ascertained by a standardized questionnaire and summed as minutes per week. Adherence to the PAG for aerobic activity was defined as engaging in at least 150 minutes a week of moderate-intensity or 75 minutes a week of vigorous-intensity aerobic physical activity or an equivalent combination of moderate- and vigorous-intensity aerobic physical activity. Time spent on sedentary behavior was estimated by hours spent sitting on a typical day.

**Results:**

Among 27 343 participants 18 years or older (13 630 [52.0%] female; 14 628 [66.6%] non-Hispanic white), the weighted adherence rate to the PAG for aerobic activity was 65.2% (95% CI, 62.3%-68.2%) in 2015-2016, with no significant change from 2007-2008 (63.2%; 95% CI, 60.2%-66.1%) to 2015-2016 (*P* = .15 for trend). Time spent on sedentary behavior significantly increased over time from a weighted mean (SE) of 5.7 (0.3) hours per day in 2007-2008 to 6.4 (0.2) hours per day in 2015-2016 (*P* < .001 for trend). The weighted proportion of people not adhering to the PAG for aerobic activity and reporting long sedentary time (>6 hours per day) increased from 16.1% (95% CI, 14.4%-17.8%) in 2007-2008 to 18.8% (95% CI, 17.7%-20.0%) in 2015-2016.

**Conclusions and Relevance:**

The findings suggest that the adherence rate to the PAG for aerobic activity in US adults has not improved since the release of the first edition in 2008 but that time spent on sedentary behavior has significantly increased over time. Further nationwide efforts appear to be warranted to not only promote physical activity but also reduce sedentary time in the United States.

## Key Points

**Question:**

What are the concurrent changing trends in adherence to the *Physical Activity Guidelines for Americans* for aerobic activity and time spent on sedentary behavior among US adults during the past decade?

**Findings:**

In a series of cross-sectional studies including data from 27 343 participants 18 years or older from the US National Health and Nutrition Examination Surveys for 2007 to 2016, the adherence rate to the *Physical Activity Guidelines for Americans* for aerobic activity was not significantly improved from 2007-2008 (63.2%) to 2015-2016 (65.2%). However, time spent on sedentary behavior increased from 5.7 hours per day in 2007-2008 to 6.4 hours per day in 2015-2016.

**Meaning:**

The findings suggest that further nationwide efforts are warranted to promote physical activity and reduce sedentary time in the United States.

## Teaser

This cross-sectional study examines the current status and secular trends in physical activity and sedentary behavior among US adults using data from the National Health and Nutrition Examination Survey (NHANES) from 2007 to 2016.

## Introduction

Insufficient physical activity has long been recognized as a risk factor for major chronic diseases and mortality.^1,2,3,4^ In 2008, the US Department of Health and Human Services released the first edition of the federal *Physical Activity Guidelines for Americans* (PAG),^5^ which was updated in 2018.^6^ The guidelines provide evidence-based guidance regarding the types and duration of physical activity in different settings to promote the public health of the general population. Specifically, both guidelines^5,6^ recommend that adults should engage in at least 150 minutes a week of moderate-intensity or 75 minutes a week of vigorous-intensity aerobic physical activity or an equivalent combination of moderate- and vigorous-intensity aerobic physical activity. Although the news about the guidelines was released by the federal government and distributed by major media outlets, a previous survey^7^ conducted in 2009 found that only 36.1% of adults reported being aware of the guidelines and less than 1% of adults were knowledgeable of the guidelines. Understanding the current status and secular changes in adherence to the guidelines over time after the release of the first edition of the guidelines is critical to inform future research and public health policy. Although several previous studies^8,9,10,11,12^ have reported the prevalence and trends of aerobic physical activity in the United States, they focused on leisure-time physical activity, possibly because of the lack of information on other domains of physical activity, including work-related activity and transportation-related activity. In a diverse population, work-related and transportation-related physical activity could account for a large proportion of aerobic physical activity for some individuals. Therefore, previous studies^9,12^ may have underestimated the true adherence rate to the PAG for aerobic activity.

In addition to engaging more in physical activity (ie, moving more), the 2018 PAG recommends that Americans reduce sedentary time (ie, sit less). Sedentary behavior has received increasing attention as a public health issue because it is prevalent in the populations and is associated with adverse health outcomes independent of physical activity levels.^13,14,15,16,17,18^ For instance, prolonged sedentary time, particularly time spent on sitting for television watching, is associated with increased risk of type 2 diabetes, cardiovascular disease, and mortality.^18^ A recent meta-analysis^19^ suggested that to attenuate or eliminate the increased risk of death associated with high sitting time, a high level of moderate-intensity physical activity (ie, approximately 60-75 minutes per day) is needed. Given the increasing evidence and recognition regarding the association of sedentary behavior with health risks, Australia^20^ and Canada^21^ have published sedentary behavior guidelines. In the United States, the 2018 PAG,^6^ for the first time, introduced sedentary behavior and the association between sedentary behavior and physical activity. Several other countries, including the United Kingdom, New Zealand, Germany, and Norway, have included sedentary behavior–related messages in their national physical activity guidelines.^22^ However, little is known about the concurrent changes in adherence to the PAG and time spent on sedentary behavior over time in the US population. In this study, we used nationally representative data from the National Health and Nutrition Examination Surveys (NHANESs) from 2007 to 2016 to estimate the temporal trends in adherence rates to the PAG^6^ for aerobic activity and time spent on sedentary behavior among US adults.

## Methods

### Study Population

This study used a series of cross-sectional data from NHANES, an ongoing, cross-sectional, nationally representative survey of the noninstitutionalized US population using a stratified, multistage probability design. Since its inception, NHANES has become a leading national survey used to assess the health and nutritional status of both adults and children. The survey collects questionnaire data on demographics, socioeconomic status, health conditions, and health-related behaviors by trained interviewers through household interviews. In addition, standardized physical examinations are performed for medical, dental, and anthropometric measurements at a mobile examination center. The NHANES is administered by the National Center for Health Statistics at the US Centers for Disease Control and Prevention; protocols of NHANES have been approved by the National Center for Health Statistics Ethics Review Board. Written informed consent was obtained from all participants before participation. The University of Iowa Institutional Review Board determined that the current study was exempt given the use of deidentified data. This study followed the Strengthening the Reporting of Observational Studies in Epidemiology (STROBE) reporting guideline.^23^

We used NHANES data from 2007-2008 through 2015-2016. The same questionnaire was used to assess physical activity during this period. We did not include data from NHANES cycles before 2007-2008 because another set of questionnaires was used to assess physical activity in those NHANES cycles. The analytic population consisted of adults 18 years or older. People without complete data on physical activity and sedentary behavior were removed from our sample. There were 27 343 individuals included in the final analytic sample.

### Data Collection

Since the 2007-2008 cycle, information on physical activity in NHANES has been self-reported by participants using the Global Physical Activity Questionnaire (GPAQ). The GPAQ is a previously validated instrument developed by the World Health Organization for physical activity surveillance.^24^ It has been applied globally in more than 100 countries through the World Health Organization Stepwise Approach to Surveillance (STEPS). The GPAQ covers several components of physical activity, such as intensity, duration, and frequency, and it assesses 3 domains in which physical activity is performed (ie, work-related physical activity, transportation-related physical activity, and physical activity during leisure time).

During the household interview, participants were asked to identify moderate- and vigorous-intensity aerobic physical activity they participated in during the past 30 days. Moderate intensity was defined as physical activity that caused small increases in breathing or heart rate and was done for at least 10 minutes continuously. Vigorous intensity was defined as physical activity that caused large increases in breathing or heart rate and was done for at least 10 minutes continuously.

The 3 domains of physical activity were measured differently. Work-related activity and leisure-time activity items queried whether the respondents participated in vigorous-intensity activity. If they answered yes, the next question inquired about the number of days in a week and minutes per day that they performed the activity. The same questions were asked for moderate-intensity activity. To estimate the amount of transportation activity (walking or bicycling to school, for shopping, or to work), questions were asked to identify the number of days in a typical week and the mean duration per day that they participated in the activity.

We calculated minutes per week of physical activity to determine whether a person met the PAG for aerobic activity. The NHANES guidelines suggest a metabolic equivalent (MET) score of 4.0 for 1 minute of transportation activity, 4.0 for moderate work-related and leisure-time activity, and 8.0 for vigorous work-related and leisure-time activity. Therefore, transportation activity was counted as moderate-intensity activity because moderate-intensity activity is defined as 3.0 to 5.9 METs.^5^ Total amount of physical activity was calculated as minutes of moderate-intensity activity plus twice the minutes of vigorous-intensity activity of all 3 domains. A person was classified as adhering to the PAG for aerobic activity if they participated in at least 150 minutes per week of moderate-intensity aerobic physical activity.

For sedentary behavior, participants were asked how much time in a typical day they spent “sitting at school, at home, getting to and from places, or with friends including time spent sitting at a desk, traveling in a car or bus, reading, playing cards, watching television, or using a computer.”^25^

Information on age, sex, race/ethnicity, ratio of family income to poverty, educational level, smoking status, and alcohol intake was ascertained by standardized questionnaires. Race/ethnicity was categorized as non-Hispanic white, non-Hispanic black, Hispanic, and others. Ratio of family income to poverty was categorized as 1.30 or less, 1.31 to 3.50, and more than 3.50. A higher income to poverty ratio represents a higher family income status. Educational level was categorized as less than high school, high school, and college or above. Participants were categorized as nonsmokers, past smokers, or current smokers based on their responses to questions about smoking at least 100 cigarettes during their lifetime and whether they were currently smoking. Alcohol intake was categorized as nondrinking (0 g/d), moderate drinking (0.1-27.9 g/d for men and 0.1-13.9 g/d for women), or heavy drinking (≥28.0 g/d for men and ≥14.0 g/d for women). Height and weight were measured by trained technicians following a standardized procedure. Body mass index was calculated as weight in kilograms divided by height in meters squared.

### Statistical Analysis

In all analyses, we followed the NHANES Analytic Guidelines^26^ to account for sample weights to obtain variance estimation in the data. Therefore, our results are generalizable to the noninstitutionalized US population. Characteristics of the study sample were weighted and are presented as means (SEs) for continuous variables or numbers (percentages) for categorical variables. According to the NHANES analytic tutorials, generalized linear models were used to compare differences in continuous variables, and χ^2^ tests were used in categorical variables.

Trends in the adherence to PAG for aerobic activity over time was tested using a multivariable logistic regression model, which included a survey cycle as an independent variable. The outcome variable was categorized as whether the participant met the PAG for aerobic activity. Covariates for the models included age group, sex, race/ethnicity, ratio of family income to poverty, educational level, smoking status, alcohol intake, and body mass index categories. The homogeneity of adherence rates across strata in each survey cycle was tested by using logistic regression models with Bonferroni correction.

Multivariable linear regression analysis was used to estimate adjusted hours per day spent on sedentary behavior across strata in each survey cycle. The homogeneity of time spent on sedentary behavior across strata in each survey cycle was tested using adjusted Wald *F* tests with Bonferroni correction. To test linear trends of sedentary behavior levels over time, we performed multivariable linear regression models with survey cycle included as an independent variable, adjusted for the aforementioned covariates. To test whether the secular trends in adherence rates or sedentary time differ across strata, we performed interaction analyses by including multiplicative terms of each stratum variable with survey cycle in the aforementioned logistic regression models or linear regression models.

All data analyses were conducted with SAS statistical software, version 9.4 (SAS Institute Inc). Two-sided *P* < .05 was considered to be statistically significant.

## Results

We included 27 343 participants 18 years or older (13 630 [52.0%] females; 14 628 [66.6%] non-Hispanic white) from NHANESs for 2007 to 2016. No significant difference was found in population distribution by age, sex, and race/ethnicity groups over time (Table 1). The weighted proportion of individuals adhering to the PAG for aerobic activity was not significantly changed, from 63.2% (95% CI, 60.2%-66.1%) in 2007-2008 to 65.2% (95% CI, 62.3%-68.2%) in 2015-2016 (*P* = .15 for trend). However, we observed significantly increased adherence rates in several subpopulations, such as female participants, non-Hispanic black participants, nonsmokers, and those who were not normal weight or obese (Table 2).

**Table 1.** Participant Characteristics by NHANES Cycle, 2007-2016^a^

Characteristic	No. (%) of Participants					*P* Value
	2007-2008 (n = 5499)	2009-2010 (n = 5893)	2011-2012 (n = 5178)	2013-2014 (n = 5498)	2015-2016 (n = 5275)	
Age, y						
18-29	1033 (21.5)	1217 (21.3)	1135 (21.3)	1156 (21.3)	1063 (20.0)	.69
30-39	848 (16.9)	873 (16.1)	821 (16.4)	857 (16.2)	837 (16.9)	
40-49	818 (19.2)	960 (18.8)	783 (17.4)	914 (17.4)	814 (16.1)	
50-64	1350 (25.0)	1389 (25.2)	1293 (26.7)	1321 (25.8)	1288 (25.7)	
≥65	1450 (17.5)	1454 (18.5)	1146 (18.3)	1250 (19.3)	1273 (21.2)	
Sex						
Male	2682 (47.9)	2863 (48.1)	2550 (48.3)	2604 (47.9)	2528 (48.0)	.07
Female	2817 (52.1)	3030 (51.9)	2628 (51.7)	2894 (52.1)	2747 (52.0)	
Race/ethnicity						
Non-Hispanic white	2544 (69.5)	2804 (68.0)	1858 (65.8)	2325 (65.8)	1103 (61.1)	.60
Non-Hispanic black	1151 (11.4)	1075 (11.4)	1396 (11.8)	1141 (11.6)	844 (12.2)	
Hispanic	1587 (13.4)	1688 (13.7)	1056 (14.5)	1245 (14.7)	1172 (17.0)	
Other	217 (5.7)	326 (6.9)	868 (7.9)	787 (7.9)	630 (9.7)	
Educational level						
Less than high school	1622 (19.6)	1610 (18.5)	1164 (16.3)	1119 (14.8)	1181 (14.0)	.01
High school	1294 (24.3)	1305 (22.5)	1014 (18.9)	1164 (20.7)	1096 (20.1)	
College or above	2298 (52.2)	2667 (55.4)	2705 (61.1)	2883 (60.7)	2743 (63.3)	
Missing	285 (3.9)	311 (3.6)	295 (3.7)	332 (3.8)	255 (2.6)	
IPR						
≤1.3	1577 (19.6)	1837 (20.5)	1801 (25.0)	1810 (23.8)	1560 (19.5)	.03
>1.3 to 3.5	1916 (32.0)	1988 (33.8)	1566 (31.4)	1746 (32.1)	1881 (34.2)	
>3.5	1491 (40.5)	1486 (37.6)	1362 (37.2)	1522 (37.6)	1283 (38.0)	
Missing	515 (7.9)	582 (8.1)	449 (6.5)	420 (6.5)	551 (8.2)	
Smoking status						
Nonsmoker	2760 (51.5)	3007 (53.5)	2793 (53.9)	3205 (57.7)	3141 (57.1)	<.001
Current smoking	1133 (21.2)	1195 (19.3)	970 (18.8)	1080 (19.3)	962 (18.2)	
Ever smoker	1322 (23.3)	1393 (23.8)	1118 (23.5)	1211 (23.0)	1165 (24.7)	
Missing	284 (3.9)	298 (3.4)	297 (3.7)	2 (0.1)	7 (0.1)	
Alcohol intake^b^						
Nondrinking	3979 (69.2)	4250 (68.1)	3629 (68.0)	3831 (67.3)	3861 (70.1)	<.001
Moderate drinking	492 (9.7)	466 (9.1)	369 (7.5)	359 (7.4)	280 (6.1)	
Heavy drinking	746 (15.9)	895 (17.8)	683 (17.3)	776 (17.7)	709 (17.3)	
Missing	282 (5.3)	282 (5.0)	497 (7.17)	532 (7.6)	425 (6.5)	
BMI categories						
Underweight	106 (1.8)	104 (2.0)	83 (1.1)	103 (1.5)	84 (1.6)	.03
Normal	1554 (31.1)	1621 (29.9)	1590 (29.8)	1622 (28.6)	1418 (27.0)	
Overweight	1832 (33.5)	1953 (33.1)	1606 (32.8)	1715 (32.1)	1639 (31.2)	
Obesity	1904 (32.0)	2145 (34.1)	1783 (34.4)	1987 (36.7)	2068 (39.0)	
Missing	103 (1.6)	70 (0.9)	83 (1.1)	71 (1.1)	66 (1.2)	

Abbreviations: BMI, body mass index; IPR, income to poverty ratio; NHANES, National Health and Nutrition Examination Survey.

^a^ Data are expressed as unweighted number of participants and weighted percentages.

^b^ Nondrinking, 0 g/d; moderate drinking, 0.1 to 27.9 g/d for men and 0.1 to 13.9 g/d for women; and heavy drinking, 28.0 g/d or more for men and 14.0 g/d or more for women.

**Table 2.** Trends in Weighted Adherence Rates to the Physical Activity Guidelines for Aerobic Activity by NHANES Cycle, 2007-2016

Characteristic	Adherence Rate, % (95% CI)^a^					*P* Value	
	2007-2008 (n = 5499)	2009-2010 (n = 5893)	2011-2012 (n = 5178)	2013-2014 (n = 5498)	2015-2016 (n = 5275)	Linear Trend^b^	Interaction^c^
Overall	63.2 (60.2-66.1)	62.7 (60.3-65.1)	64.9 (61.6-68.3)	61.4 (59.5-63.3)	65.2 (62.3-68.2)	.15	NA
Age, y							
18-29	75.1 (71.6-78.7)	74.0 (69.6-78.4)	79.7 (74.7-84.7)	73.7 (70.6-76.8)	79.1 (76.3-81.9)	.14	.08
30-39	70.5 (66.7-74.2)	66.7 (60.4-73.0)	70.9 (67.0-74.8)	69.9 (65.4-74.5)	74.7 (69.4-80.1)	.05	
40-49	62.9 (58.8-67.0)	67.4 (64.6-70.3)	65.6 (60.6-70.7)	64.4 (59.6-69.3)	67.0 (61.1-73.0)	.13	
50-64	61.3 (55.8-67.0)	60.9 (57.1-64.6)	61.3 (57.0-65.7)	56.7 (52.4-61.0)	60.4 (56.6-64.2)	.50	
≥65	44.3 (38.7-49.8)	43.9 (39.6-48.2)	46.8 (43.5-50.2)	44.4 (39.2-49.6)	49.1 (43.5-54.7)	.57	
*P* value for age^d^	<.001	<.001	<.001	<.001	<.001	NA	NA
Sex							
Male	71.7 (68.7-74.6)	71.5 (69.0-74.1)	71.9 (68.3-75.4)	68.6 (65.5-71.7)	72.0 (69.9-74.2)	.75	.09
Female	55.3 (51.6-59.0)	54.5 (51.6-57.5)	58.4 (54.4-62.3)	54.9 (52.5-57.2)	59.0 (54.5-63.4)	.04	
*P* value for sex^d^	<.001	<.001	<.001	<.001	<.001	NA	NA
Race/ethnicity							
Non-Hispanic white	65.8 (61.6-69.9)	65.1 (61.8-68.5)	67.3 (63.2-71.4)	60.6 (57.7-63.6)	67.6 (64.7-70.4)	.70	.08
Non-Hispanic black	52.7 (46.9-58.6)	56.8 (53.6-60.1)	61.3 (56.9-65.6)	60.4 (56.8-63.9)	62.6 (59.1-66.1)	<.001	
Hispanic	59.7 (53.8-65.6)	59.3 (54.3-64.3)	57.9 (53.6-62.2)	65.1 (62.8-67.3)	60.3 (56.1-64.6)	.13	
Other	60.3 (52.6-68.0)	55.0 (48.9-61.1)	63.6 (58.2-69.1)	62.7 (59.1-66.3)	60.6 (54.8-66.4)	.45	
*P* value for race^d^	<.001	<.001	<.001	.79	<.001	NA	NA
Educational level							
Less than high school	53.3 (48.7-57.8)	51.4 (47.4-55.3)	50.8 (46.0-55.6)	54.3 (49.4-59.2)	49.4 (44.2-54.6)	.50	.97
High school	60.6 (57.0-64.2)	59.6 (55.7-63.6)	61.6 (55.4-67.8)	56.9 (52.9-61.0)	61.8 (57.4-66.2)	.50	
College or above	67.2 (63.8-70.6)	67.2 (63.6-70.9)	68.7 (64.9-72.6)	63.7 (61.1-66.3)	69.3 (66.6-72.1)	.05	
*P* value for educational level^d^	<.001	<.001	<.001	<.001	<.001	NA	NA
IPR							
≤1.3	56.4 (51.1-61.8)	58.5 (54.8-62.3)	61.2 (55.1-67.3)	57.8 (55.3-60.3)	59.0 (54.1-63.8)	.34	.84
>1.3 to 3.5	59.9 (56.3-63.5)	59.8 (55.2-64.5)	65.1 (60.2-70.1)	60.9 (57.2-64.7)	63.9 (60.7-67.2)	.07	
>3.5	69.3 (65.8-72.8)	69.7 (66.8-72.6)	68.4 (63.4-73.4)	65.0 (60.6-69.3)	71.3 (67.8-74.8)	.57	
*P* value for IPR^d^	.21	.14	.96	.65	.88	NA	NA
Smoking status							
Nonsmoker	61.8 (58.2-65.4)	63.2 (59.7-66.7)	65.5 (60.6-70.4)	62.8 (60.6-64.9)	65.4 (61.4-69.4)	.03	.73
Current smoking	63.9 (59.7-68.1)	63.6 (60.3-66.9)	61.5 (58.7-64.3)	61.6 (57.3-66.0)	68.4 (62.9-73.9)	.90	
Ever smoker	63.3 (60.4-66.2)	59.7 (54.6-64.7)	63.7 (59.0-68.3)	58.0 (54.0-62.0)	62.4 (58.8-66.1)	.39	
*P* value for smoking^d^	.03	.04	.01	.55	.40	NA	NA
Alcohol intake^e^							
Nondrinking	60.6 (57.9-63.3)	58.2 (55.1-61.3)	62.8 (59.7-65.9)	60.2 (58.2-62.3)	62.4 (59.8-64.9)	.05	.01
Moderate drinking	68.2 (62.2-74.2)	75.6 (70.9-80.2)	72.0 (66.5-77.5)	69.2 (61.3-77.2)	78.8 (73.6-84.0)	.11	
Heavy drinking	71.6 (66.8-76.5)	73.5 (69.0-78.1)	71.8 (66.2-77.4)	68.0 (63.9-72.2)	76.5 (71.0-81.9)	.17	
*P* value for alcohol^d^	.26	.05	.26	.04	.14	NA	NA
BMI categories							
Underweight	62.7 (49.6-75.7)	61.6 (51.4-71.8)	65.4 (50.2-80.6)	56.5 (41.1-71.9)	58.5 (46.7-70.3)	.60	.41
Normal	68.5 (63.9-73.0)	68.3 (63.7-73.0)	71.7 (67.4-76.1)	69.7 (67.4-72.1)	72.3 (68.0-76.6)	.04	
Overweight	66.8 (64.7-68.8)	64.9 (61.9-67.9)	67.9 (64.8-71.1)	64.7 (60.9-68.5)	65.4 (60.9-70.0)	.98	
Obesity	55.4 (51.6-59.2)	56.2 (53.1-59.3)	57.0 (52.6-61.3)	53.6 (50.8-56.4)	61.5 (58.3-64.7)	.03	
*P* value for BMI^d^	<.001	<.001	<.001	<.001	<.001	NA	NA

Abbreviations: BMI, body mass index; IPR, ratio of family income to poverty; NA, not applicable; NHANES, National Health and Nutrition Examination Survey.

^a^ Data are presented as percentage (95% CI) and are weighted.

^b^ Multivariable logistic regression models include NHANES cycle as a continuous term adjusted for all covariates.

^c^ *P* values for interaction between socioeconomic variables and trend variable in multivariable logistic regression analyses adjusted for all covariates.

^d^ *P* values for homogeneity estimated with Bonferroni correction for multiple comparisons in multivariable logistic regression analyses adjusted for all covariates.

^e^ Nondrinking, 0 g/d; moderate drinking, 0.1 to 27.9 g/d for men and 0.1 to 13.9 g/d for women; and heavy drinking, 28.0 g/d or more for men and 14.0 g/d or more for women.

The time spent on sedentary behavior increased significantly from a weighted mean (SE) of 5.7 (0.3) hours per day in 2007-2008 to 6.4 (0.2) hours per day in 2015-2016 (*P* < .001 for trend). The 2013-2014 cycle had the highest sedentary time (adjusted mean [SE], 7.9 [0.4] hours per day). The increase in sedentary time was observed in almost every subgroup evaluated. In addition, the time spent on sedentary behavior was the highest among people with educational levels of college or above and in the obese population (Table 3).

**Table 3.** Trends in Weighted Mean Hours per Day of Sedentary Behavior by NHANES Cycle, 2007-2016

Characteristic	Adjusted Mean (SE)^a^					*P* Value	
	2007-2008 (n = 5499)	2009-2010 (n = 5893)	2011-2012 (n = 5178)	2013-2014 (n = 5498)	2015-2016 (n = 5275)	Linear Trend^b^	Interaction^c^
Overall^a^	5.7 (0.3)	5.9 (0.2)	6.0 (0.2)	7.9 (0.4)	6.4 (0.2)	<.001	NA
Age, y							
18-29	5.8 (0.2)	6.0 (0.2)	6.1 (0.3)	7.9 (0.5)	6.5 (0.2)	<.001	.01
30-39	5.5 (0.3)	6.1 (0.2)	5.9 (0.2)	7.8 (0.4)	5.8 (0.3)	<.001	
40-49	5.4 (0.3)	5.5 (0.2)	6.0 (0.2)	7.9 (0.4)	6.2 (0.3)	<.001	
50-64	5.8 (0.3)	5.9 (0.2)	6.0 (0.2)	7.9 (0.4)	6.1 (0.2)	<.001	
≥65	6.0 (0.3)	5.9 (0.2)	6.1 (0.2)	7.9 (0.4)	6.0 (0.3)	<.001	
*P* value for age^d^	.003	.007	.59	.89	.02	NA	NA
Sex							
Male	5.7 (0.3)	6.0 (0.2)	6.1 (0.2)	7.9 (0.4)	6.2 (0.2)	<.001	.64
Female	5.7 (0.3)	5.8 (0.2)	6.0 (0.2)	7.9 (0.4)	6.1 (0.3)	<.001	
*P* value for sex	.90	.07	.39	.50	.58	NA	NA
Race/ethnicity							
Non-Hispanic white	5.9 (0.2)	6.2 (0.2)	6.3 (0.2)	8.0 (0.5)	6.6 (0.2)	<.001	.08
Non-Hispanic black	5.8 (0.2)	5.8 (0.2)	6.4 (0.3)	8.5 (0.4)	6.3 (0.2)	<.001	
Hispanic	5.0 (0.3)	5.0 (0.2)	5.0 (0.2)	7.1 (0.4)	5.4 (0.3)	<.001	
Other	6.0 (0.5)	6.5 (0.2)	6.3 (0.2)	8.0 (0.4)	6.2 (0.2)	.03	
*P* value for race^d^	<.001	<.001	<.001	<.001	<.001	NA	NA
Educational level							
Less than high school	4.6 (0.3)	5.1 (0.2)	5.5 (0.3)	7.5 (0.4)	5.5 (0.2)	<.001	<.001
High school	4.9 (0.3)	5.3 (0.2)	5.6 (0.4)	7.7 (0.4)	6.0 (0.2)	<.001	
College or above	5.8 (0.3)	6.1 (0.2)	6.5 (0.3)	8.2 (0.4)	6.5 (0.2)	<.001	
*P* value for educational level^d^	<.001	<.001	<.001	<.001	<.001	NA	NA
IPR							
≤1.3	5.3 (0.3)	5.7 (0.2)	5.6 (0.2)	7.6 (0.4)	6.0 (0.3)	<.001	.07
>1.3-3.5	5.6 (0.2)	5.7 (0.2)	5.8 (0.3)	7.6 (0.4)	6.2 (0.3)	<.001	
>3.5	6.4 (0.3)	6.5 (0.3)	6.6 (0.2)	8.3 (0.4)	6.5 (0.2)	<.001	
*P* value for IPR^d^	<.001	.56	<.001	<.001	.03	NA	NA
Smoking status							
Nonsmoker	6.0 (0.2)	6.1 (0.3)	6.0 (0.3)	7.4 (0.2)	6.2 (0.2)	<.001	.51
Current smoking	6.0 (0.3)	6.4 (0.4)	6.2 (0.3)	7.2 (0.3)	6.0 (0.2)	<.001	
Ever smoker	6.2 (0.3)	6.2 (0.3)	6.2 (0.2)	7.4 (0.2)	6.2 (0.2)	<.001	
*P* value for smoking^d^	.01	.11	.68	.45	.52	NA	NA
Alcohol intake^e^							
Nondrinking	5.5 (0.3)	5.9 (0.2)	6.2 (0.2)	7.8 (0.4)	6.1 (0.2)	<.001	.06
Moderate drinking	5.9 (0.3)	6.0 (0.3)	5.9 (0.3)	7.7 (0.5)	5.8 (0.3)	.12	
Heavy drinking	5.6 (0.3)	5.8 (0.2)	5.8 (0.2)	7.9 (0.4)	6.1 (0.2)	<.001	
*P* value for alcohol^d^	.46	.68	.05	.25	.24	NA	NA
BMI categories							
Underweight	5.1 (0.4)	5.7 (0.4)	5.5 (0.5)	7.6 (0.5)	5.1 (0.5)	0.23	.84
Normal	5.1 (0.2)	5.0 (0.2)	5.5 (0.2)	7.3 (0.5)	5.6 (0.2)	<.001	
Overweight	5.4 (0.2)	5.3 (0.1)	5.8 (0.2)	7.4 (0.4)	5.9 (0.2)	<.001	
Obesity	5.8 (0.2)	5.8 (0.2)	6.2 (0.1)	8.0 (0.4)	6.4 (0.2)	<.001	
*P* value for BMI^d^	<.001	<.001	.01	.003	<.001	NA	NA

Abbreviations: BMI, body mass index; IPR, income to poverty ratio; NA, not applicable; NHANES, National Health and Nutrition Examination Survey.

^a^ Data are presented as adjusted means (SEs) estimated by multivariable linear regression analysis, adjusted for all covariates except stratification, and are weighted.

^b^ Multivariable linear regression models include NHANES cycle as a continuous term adjusted for all covariates.

^c^ *P* values for interaction between socioeconomic variables and trend variable in multivariable linear regression analyses adjusted for all covariates.

^d^ *P* values for homogeneity estimated by the adjusted Wald *F* test with Bonferroni correction for multiple comparisons in multivariable linear regression analyses adjusted for all covariates.

^e^ Nondrinking, 0 g/d; moderate drinking, 0.1 to 27.9 g/d for men and 0.1 to 13.9 g/d for women; and heavy drinking, 28.0 g/d or more for men and 14.0 g/d or more for women.

We also estimated the trends in joint prevalence of physical inactivity and long sedentary time (Figure). The weighted proportion of adults who did not meet the PAG for aerobic activity but spent over 6 hours per day on sedentary behavior increased significantly from 16.1% (95% CI, 14.4%-17.8%) in 2007-2008 to 18.8% (95% CI, 17.7%-20.0%) in 2015-2016 (*P* < .001 for trend), and the estimated proportion was the highest in the 2013-2014 cycle (25.9%; 95% CI, 23.9%-27.8%).

**Figure.**
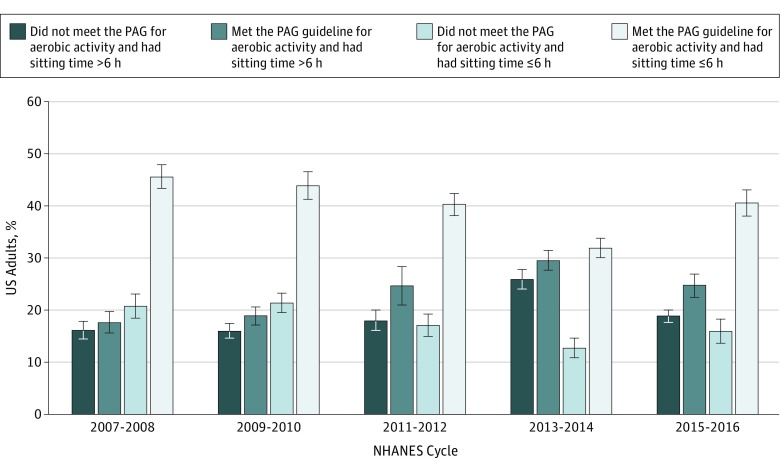
Trends in the Joint Distribution of Aerobic Physical Activity and Sedentary Time Among US Adults, National Health and Nutrition Examination Survey (NHANES), 2007-2016 Data are weighted percentage of US adults in each category. Error bars indicate 95% CIs. PAG indicates Physical Activity Guidelines for Americans.

## Discussion

In a nationally representative survey of US adults, we found that the proportion of people meeting the PAG for aerobic activity was 65.2% in 2015-2016, and the adherence rate was not significantly improved from 2007-2008 through 2015-2016. In contrast, there was a significant increase in time spent on sedentary behavior over time.

Aerobic physical activity contains multiple domains, including leisure-time, work-related, and transportation-related aerobic activity. The PAG for aerobic activity refers to all domains of aerobic activity. Our study for the first time, to our knowledge, examined the national trends in adherence rates to the PAG for aerobic activity based on all domains (leisure time, work related, and transportation related) of aerobic activity in US adults. Several studies^8,9,10,11,12^ have reported the prevalence of and/or trends in the adherence to the PAG in the United States. However, the reported adherence rates in those studies were based only on the leisure-time domain of aerobic activity. Therefore, the reported adherence rates (eg, 49%^9^ and 44.6%^12^) in previous studies, which were lower than the estimates in our study, could not reflect the contribution of work-related and transportation-related aerobic activity. Of note, direct comparison in the estimated rates between our study and previous studies^8,9,10,11,12^ might not be meaningful because of this important conceptual difference (ie, all 3 domains vs leisure-time domain only).

The adherence rates to the PAG for aerobic activity varied with age, with the oldest adults having the lowest adherence rates in each survey cycle. This finding may be attributable to the decreased aerobic capacity and/or the presence of illness and disability among older adults. People with higher educational levels had higher adherence rates in each cycle and had an increased trend in recent years. This finding is consistent with previous findings that people with higher educational levels might purposefully choose to do more exercise during their leisure time to enhance their health.^27,28^ Findings on the racial/ethnic and sex differences were also consistent with previous results.^8,9,29,30^ Findings from the National Health Interview Survey indicated that among adults, men and non-Hispanic white individuals had a greater level of physical activity during leisure time.^8,9,29^ A previous report^30^ using objective measurements also found that females had lower physical activity levels. Furthermore, in the current study, non-Hispanic black persons and females tended to have increased adherence rates to the PAG for aerobic activity during the past 10 years. Future approaches toward increasing physical activity levels should consider addressing sex- and race/ethnicity-specific barriers.

We observed a significant increase in time spent on sedentary behavior during the past 10 years, and the increase was consistent across the subgroups examined. The sedentary time was the highest in the 2013-2014 cycle; the reasons for this remain unclear and warrant further investigation. These findings were consistent with a recent study.^31^ Studies^13,14,15,16,17,18^ have found associations between sedentary behaviors and increased risk of a wide range of health outcomes, including all-cause mortality, cardiovascular disease, cancer, obesity, and an adverse metabolic profile. Significant associations between obesity and sedentary behavior were also observed in our study. There is evidence that obesity and severe obesity prevalence increased among adults between 2007-2008 and 2015-2016.^32^ Although the current study design does not permit identification of the casual association between change in sedentary behavior and change in obesity during this study period, interventions that target sedentary behaviors may need to be considered for controlling the obesity epidemic.

Our study has significant public health implications. Both insufficient physical activity and prolonged sedentary time are associated with a high risk of adverse health outcomes, including chronic diseases and mortality.^1,2,3,4,13,14,15,16,17,18^ Sedentary behavior is not just the inverse of physical activity.^33^ As shown in a recent meta-analysis,^19^ high volumes of moderate to vigorous physical activity are required to attenuate the excess risk of high sedentary time–associated mortality. Therefore, our findings highlight a critical need for future public health efforts to aim for not only an increase in physical activity but also a reduction of sedentary time. Given the high volume of sitting time and low levels of physical activity in the general population, most people may benefit from both engaging in moderate to vigorous physical activity and reducing time spent sitting. As stated in the 2018 PAG, “Adults should move more and sit less throughout the day. Some physical activity is better than none. Adults who sit less and do any amount of moderate to vigorous physical activity gain some health benefits.”^6^^(p 56)^

### Strengths and Limitations

Our study has several strengths. First, NHANES is a nationally representative survey of the noninstitutionalized US population; thus, the study population is representative of the US general population. Second, the abundant data on population characteristics allowed us to estimate the physical activity levels in specific subgroups. Third, the data collection in NHANES was conducted under robust quality assurance and control procedures, which improves the reliability of the data.

A major limitation of this study is that the information on physical activity was self-reported, which may be subject to misreporting and recall bias. Second, the questionnaire asked for each type of physical activity with a duration of at least 10 minutes; therefore, we were unable to capture physical activity episodes shorter than 10 minutes. Third, although the PAG also includes recommendations regarding muscle-strengthening activities, we were unable to address the trends of muscle-strengthening activities in this study because information about muscle-strengthening activities among adults was not assessed in NHANES 2007-2016.

## Conclusions

The findings suggest that, since the release of the first PAG in 2008, there has been no significant increase in the percentage of individuals adhering to the PAG for aerobic activity; however, time spent on sedentary behaviors significantly increased. Future nationwide efforts may be warranted to promote physical activity and reduce sedentary time in US adults.
